# The effectiveness of the post-caesarean section home care guide in increasing the knowledge of nurse midwives in central Tanzania

**DOI:** 10.3389/fgwh.2026.1697841

**Published:** 2026-06-16

**Authors:** Mwajuma Bakari Mdoe, Stephen Mathew Kibusi, Lilian Teddy Mselle

**Affiliations:** 1 Department of Clinical Nursing, School of Nursing and Public Health, University of Dodoma, Dodoma, Tanzania; 2 Department of Clinical Nursing, School of Nursing, Muhimbili University of Health and Allied Sciences, Dar es Salaam, Tanzania; 3 Department of Public Health, School of Nursing and Public Health, University of Dodoma, Dodoma, Tanzania

**Keywords:** discharge education, education intervention, home care after caesarean section, nurse midwive's knowledge, post-caesarean section home care guide

## Abstract

**Background:**

Evidence suggests that nurse midwives often provide unsatisfactory health education to post-caesarean (CS) mothers regarding home care. The increase in CS has led to the premature discharge of mothers with insufficient guidance, which may result in an abnormal recovery. This might be due to the lack of a home care guide to educate mothers on how to care for themselves at home after discharge. The authors developed a post-caesarean section home care guide and assessed its effectiveness at increasing knowledge of home care among nurse midwives working in a maternity unit in central Tanzania.

**Methods:**

This quasi-experimental study involved 126 nurse midwives working in a maternity unit of Dodoma and Singida regional Hospital. Participants were assessed on their level of knowledge of home care after CS both before and after they were exposed to the intervention. The intervention involved familiarizing the nurse midwives to the developed home care guide. SPSS Version 27 was used in data analysis.

**Results:**

The mean knowledge score was 20.29 (±4.036) and 24.59 (SD ± 3.338) before and after the intervention, respectively, and the difference was statistically significant. Linear regression reported the intervention’s large effect size (Cohen's *d* = 1.26). Education intervention was significantly associated with improved knowledge of home care after hospital discharge (*β* = 6.401, t = 2.856, R2 = 0.257, *p*-value = 0.005, CI: 1.959–10.844). By making the intervention constant, no other factor contributed to the change.

**Conclusion:**

The knowledge of nurse midwives on home care after caesarean section is generally low. Providing nurse-midwives with the developed home care guide improves their understanding and ensures the delivery of standardized education. This supports a safe recovery for mothers, which is particularly vital given current high cesarean section rates.

## Introduction

The postpartum period is an important period but also when the majority of complications to both mother and baby occur. The postpartum period especially following caesarean section (CS) is a significant period where mothers are at increased risk of complications such as surgical site infections (SSI). Tanzania has the highest rate of SSI, occurring in up to 48% of cases, and most maternal deaths occur after CS (1); this reflects the urgent need for effective strategies to improve post-discharge care and prevent complications. Enhanced recovery after CS involves the early discharge of post-CS from 48 h (2) and, due to the current increase in CS rates, women are being discharged even earlier to prevent congestion on post-CS wards. Strong home care support, especially for mothers returning to an under-resourced environment, may reduce the risk of complications. Hence, evidence-based, structured home care education is important in increasing the awareness of post-CS mothers and their confidence in home care to achieve smooth recoveries (3).

Nurse midwives are crucial to ensuring quality care is provided to both mother and the; they also play a pivotal role in ensuring safe post-CS care by educating mothers on home care after CS. Despite their important role, nurse midwives' education is constrained due to systematic challenges, including time limitations and a lack of standardized training material (4, 5), which highlights the importance of on-the-job training and standardized clinical care protocols.

On-the-job training has been thought to be an effective approach for improving nurse-midwives’ knowledge and practice. Evidence from various low and middle income countries reveals that structured training and educational interventions lead to improvements in clinical skills and knowledge. For example, significant improvement of knowledge (from 77.5% to 93%) and skills (from 37.5% to 83%) of nurse midwives has been reported from the “Helping Mother's Survive Bleeding After Birth” program in Tanzania, with high retention at 10 months. Similarly, the Safe Delivery Application (SDA) tool in Rwanda, a mobile learning tool delivering evidence-based obstetric content, was associated with measurable gains in postpartum hemorrhage management and neonatal resuscitation (21). These findings underscore the potential of structured, context-specific educational interventions to enhance clinical competencies and improve patient outcomes. Evidence shows that no or non-uniform information is provided to post-CS mothers by nurse midwives on home care after CS (6). This might be due to the lack of a post-CS home care guide that has comprehensive, structured home care information tailored to the needs of women recovering from CS in Tanzania. Rwanda has developed a technical consensus guideline for post-discharge care, although some recommendations are not applicable to the Tanzanian context, like changing gauze at home after bathing.

A clear, context-specific guide with updated content may help provide standardized health education, preventing the delivery of conflicting or confusing information (3). A home care guide might help provide clear communication on what to educate mothers on after being discharged from the hospital, especially in developing countries like Tanzania (3). Further, evidence-based and structured discharge education helps raise awareness of potential complications and safe recovery practices for women and their families. Authors have developed the home care guide for nurse midwives to provide uniform and reliable education to post-CS mothers on how to care for themselves at home after CS in Tanzania (7). In this study, a home care guide refers to a structured instructional material developed to provide evidence-based information and practical recommendations to nurse midwives regarding post-CS home care. The guide took into account the low economic status found in developing countries. The introduction of a post-CS home care guide is a strategic intervention aimed at equipping nurse-midwives with the necessary knowledge to support patients effectively during the recovery period. By providing a clear, evidence-based guide, this educational tool is expected to improve the competency of nurse-midwives in central Tanzania, thereby enhancing the overall quality of post-CS care and potentially reducing complications and readmissions. This study aims to assess the effectiveness of the post-CS home care guide in increasing knowledge of home care after hospital discharge among nurse midwives working in the maternity unit in central Tanzania, which is an essential step in justifying its use.

## Methodology

### Study design

This was a quasi-experimental study where knowledge on home care after hospital discharge was assessed in nurse midwives before and after they received the intervention. The design was suitable as it helps to compare the before and after intervention knowledge necessary for evaluating the effectiveness of the guide in increasing nurse midwives’ knowledge on post-CS home care.

### Study area

This study was conducted in referral hospitals in the Dodoma and Singida regions, Tanzania. In Dodoma, the study was conducted at Dodoma Regional Referral Hospital (DRRH) and, in Singida, the study was conducted at Singida Regional Referral Hospital (SRRH). DRRH and SRRH were chosen because they have higher rates of SSI after CS (8) and CS deliveries (7). Both hospitals are located in central Tanzania, provide Comprehensive Emergency Obstetrics and Newborn Care (CEmONC), and have separate maternity blocks.

### Study population

This study recruited 126 nurse midwives, 63 from DRRH and 63 SRRH. The nurse midwives were from maternity wards including post-CS wards. Maternity ward nurses typically work in rotations; however, patients require consistent, interactive care to improve their recovery and long-term outcomes. In both hospitals, the nurses were from antenatal wards, labor wards, postnatal wards, gynecological wards, and post-CS wards. A convenient sampling strategy was used for selecting nurse midwives.

The sample size for the nurses and midwives was obtained using the formula for comparing two dependent samples; nurse knowledge on the timing of administration of uterotonics before intervention was 44.8% and after educational intervention was 63.4% (9).

n=(Zα+Zβ)2/(E/S)2

where Zα = The Z-score for the significance level (1.96)

Zβ = The Z-score for the desired power of the study (80% = 0.8).

The expected Effect size is 0.13.

S = Standard deviation of outcome variable, at 95% (0.5).

n=(1.96+0.8)2/(0.13/0.5)2

n=112.6≈113

Plus 10% attrition rate = 125 minimum sample size.

Since this study involved nurse midwives from two hospitals, we included 126 nurses, 63 from each hospital.

### Intervention

Preparing for the intervention involved setting up the venue, connecting the laptop to the projector, printing the home care guides, and booking three sessions for each hospital. The first author and research assistant led the interventions for nurse midwives in both hospitals. Before the intervention, nurse midwives were assessed on their baseline knowledge on home care after CS by using a pre-tested questionnaire. The pre-test took 20 to 30 min, thereafter, nurse midwives were trained on the content of the guide and how they would use the guide to educate post-CS mothers. The training involved the presentation of all components of the guide through a lecture and slide projection in the meeting room at DRRH and SRRH. The components of the guide included nutrition, wound care, exercise, hygiene, family planning, postnatal service utilization, sexual activities, and mental health care. Nurse midwives were also provided with the leaflet for the guide for proper follow up.

In all hospitals, the intervention involved six training sessions scheduled on six different days for both hospitals, since it is difficult to get all of the nurse midwives in one day. The intervention took one hour. After the intervention, group discussion followed, where nurse midwives were allowed to ask questions to enhance their understanding. Subsequently, they were tested on the same day. The nurse midwives working in postnatal wards were required to provide home care education intervention to post-CS mothers to ensure the intervention is sustainable.

### Data collection tool and method

A semi-structured questionnaire was used for data collection. The tool was developed with regard to the designed home care guide for mothers after CS. The tool was pre-tested at Morogoro Regional Referral Hospital to test its validity and reliability in measuring the knowledge of nurse midwives regarding home care after hospital discharge. The tool consisted of the following: demographic characteristics; experience with caring for post-CS mothers; and questions on the home care of the mother regarding nutrition, wound care, exercise, hygiene, family planning, postnatal service utilization, sexual activities, and mental health care. The tool was developed in English and administered to the nurse midwives in the form of a hard copy to make collecting responses easier. Nurse midwives were assessed in the meeting room to offer them a comfortable environment. Both pre-assessment and post-assessment took about 20–30 min.

### Validity and reliability of the tool

Following the pre-test, data analysis was performed using SPSS version 27. Data cleaning and completeness were assessed by running the frequency of items per (10) variable. Four questions were removed from the data set during manual cleaning because they had no variance. Thirty-six questions were subjected to axploratory factor analysis, set at KMO ≥ 0.5 and a significance level of ≤0.05. Weight per item was set at ≥0.3, as recommended by previous scholars (10). Initially, all items weighed above ≥0.3 and therefore were retained for further analysis.

To ensure the internal reliability of the tool, scale analysis was done on 36 items, and findings showed α = 0.448 with four items having zero or approximately zero covariance; these items were removed and not included in the second analysis. The second analysis involved 32 items and the α = 0.509. No item had zero or approximately zero covariance; hence all 32 items were retained for further analysis.

### Measurement of variable

The knowledge of the nurse midwives was measured numerically.

### Analysis

Data analysis was performed using SPSS V.27. The collected data were entered into the computer and checked for cleanness as well as missing data. Descriptive analysis was performed to understand the distribution of participants by demographic characteristics, estimate prevalence, and describe patterns of home care knowledge. Component factor analysis was employed for data reduction purposes to establish the weight of each item and allow for inclusion when computing the final composite variable on individual scores on the knowledge of participants on home care after CS. To test the relationship between the independent variables and the continuous dependent variable of knowledge score, linear regression analysis was used. A 95% CI with a 5% margin of error (0.05) was used as the statistical measure of significance. Before that, the Q-Q plot was used to test the normality distribution of outcome knowledge; the results showed normal distribution of the knowledge score.

### Validity and reliability of the study

The sample size of 126 was sufficient to represent the population of nurse midwives working in maternity units in Central Tanzania. The use of the pretested tool at Morogoro Regional Referral Hospital ensured the tool measured what was intended. The tool was also reviewed by maternal and child health experts and statisticians to ensure the correct content of home care questions relevant to the guide, the order of questions, and the clarity and consistency of ideas.

### Ethical approval

The Institutional Review Board at Muhimbili University of Health and Allied Sciences approved our interviews (approval: REC-05-2021-647) on 22nd April, 2024. Respondents gave their written consent for review before starting data collection, after they were explained about the aim of the study, procedures, and their freedom to withdraw from the study at any time.

## Results

### Socio-demographic characteristics of nurse midwives

This study involved 126 nurse midwives working on a maternity ward in the central zone of Tanzania and had an 100% response rate. As shown in table one, 50% (*n* = 63) of nurses were from SRRH and 50% (*n* = 63) were from DRRH. The majority of nurse midwives (68.3%) were female and aged (55.7%) between 23 and 35 years and with a diploma level (54.8%) of training. The majority of nurse midwives had 5–10 years of experience (29.4%); 34.9% were from a post-CS ward, as shown in Table 1.

**Table 1:** Demographic characteristics of nurse midwives (*N* = 126).

Variable	Frequency (*n*)	Percentage (%)
Sex		
Male	40	31.7
Female	86	68.3
Age: 36.17 ± 9.16		
23–35	70	55.6
36–49	45	35.7
50–60	11	8.
Highest level of training		
Certificate	30	23.8
Diploma	69	54.8
Bachelor and above	27	21.4
Years since Training		
1–2 years	18	14.3
3–5 years	36	28.6
6–10 years	37	29.4
>10 years	35	27.8
Ward		
Antenatal ward	16	12.7
Labor ward	39	31.0
Post-natal wad	27	31.4
Post-CS ward	44	34.9

### Mean score difference in knowledge of nurse midwives before and after the intervention

The mean knowledge score before intervention was 20.29 (±4.036) and the mean knowledge score after intervention was 24.59(SD ± 3.338); and the difference is statistically significant. Cohen's d formula was used to determine the effect size of the intervention on nurse midwives’ knowledge of home care after CS before and after the intervention (11). Findings demonstrate that the intervention has large effect size (small = 0.2, medium = 0.5, large = 0.8). This indicates a substantial difference between the pre-interventional and post-interventional knowledge scores, suggesting that the intervention had a strong impact on home care knowledge, as shown in Table 2.

**Table 2:** Paired *t*-test results.

Pair sample test	Mean	SD	Std. error mean	Confidence Interval		*t*	df	*P*-Value	*Effect size* $Cohen^{\prime }s\;d=\frac{\left(M2-M1\right)}{SDpooled}$
				Lower	Upper				
Knowledge 1	20.29	4.036		19.58	21.01				
Knowledge 2	24.56	3.338		23.97	25.15				
Knowledge 2-knowledge 1	4.270	4.703	0.419	3.441	5.099	10.191	125	˂0.001	1.26

*Knowledge 1 = Pre-interventional knowledge.

*Knowledge 2 = Post-interventional Knowledge.

### Level of knowledge on home care among nurse midwives

For descriptive purposes, the knowledge level of nurse midwives was categorized based on the mean score. As shown in Table 3, the baseline level of knowledge among nurse midwives on home care after CS was low: 52.4% (*n* = 66) had a high level of knowledge before the intervention while 95.2% (*n* = 120) had a high level of knowledge after (Table 3).

**Table 3:** Prevalence of knowledge on home care among nurse midwives (*N* = 126).

Knowledge	Frequency (*n*)	Percentage (%)
Baseline knowledge		
Low level of understanding	66	52.4
High level of understanding	60	47.6
Post-interventional knowledge		
Low level of understanding	6	4.8
High level of understanding	120	95.2

### Effectiveness of the guide in improving knowledge of nurse midwives

Linear regression analysis showed that the 1 h of teaching nurse midwives to post-CS home care guide was significantly associated with improved knowledge on home care after hospital discharge (β = 6.401, t = 2.856, R2 = 0.257, *p*-value, 0.005 CI: 1.959, 10.844). This indicates that, after controlling for other factors, nurse midwives who received the home care education intervention scored an average of 6.04 units higher on knowledge of home care after CS compared to their counterparts. The association is statistically significant and not associated with other factors, as shown in Table 4.

**Table 4:** Linear model between demographic characteristics and knowledge of nurse midwives on home care after CS.

Variables	*β*	Coefficient of standard error	*t*	*P*-value	95% CI		*R* ^2^
					Lower value	Upper value	
Sites							0.257
Baseline	Ref						
Endline	6.401	2.242	2.856	0.005	1.959	10.844	
Age							
23–35 years	Ref						
26–49 years	0.458	1.153	0.397	−0.692	1.827	2.743	
50–60 years	0.538	2.015	0.267	−0.790	3.455	4.530	
Sex							
Male	Ref						
Female	0.576	0.997	0.057	−0.955	0.392	2.033	
Highest level of training							
Certificate	Ref						
Diploma	0.046	1.297	0.036	−0.972	0.350	2.162	
Bachelor and above	1.020	1.188	0.858	−0.393	0.183	3.108	
Professional Experience							
1–2 years	Ref						
3–5 years	0.837	1.519	0.551	−0.583	0.755	1.737	
6–10 years	0.386	1.204	0.320	−0.749	0.864	2.798	
>10 years	0.730	1.518	0.481	−0.631	0.163	1.442	
Ever trained on Post-CS home care?							
No	Ref						
Yes	0.015	0.928	0.016	−0.987	0.213	2.771	
Ward							
Antenatal	Ref						
Labor ward	0.471	1.160	0.406	−0.685	0.828	4.770	
Post-SVD ward	0.383	1.299	0.295	−0.768	0.191	1.958	
Post-CS ward	1.246	1.494	0.834	−0.406	1.014	4.206	

## Discussion

This study assessed the effectiveness of the post-CS home care guide in improving the knowledge of nurse midwives. In this study, the majority of nurse midwives were female, reflecting historical perceptions of nursing as a female profession, although recent studies indicate a growing number of men entering the field (12–14). More than half of nurse midwives (55.6%) were aged 23–35 years, a finding similar to those from Egypt (Hussien et al. 2021) and the Tanzania Demographic and Health Survey (TDHS, 2022), which report high employment levels in this age group. Most participants had a diploma-level education, differing from Joho et al. (9), where certificate-level training predominated. This discrepancy might be due to the rural setting of that study versus. the urban focus of the present study, where education levels are generally higher (TDHS, 2022). Moreover, 57.2% had over five years of work experience, comparable to findings by Joho et al. (9), indicating that participants possessed substantial professional experience relevant to the study topic.

In this study, we found a large effect size of the post-CS home care guide in increasing the knowledge of home care after CS among nurse midwives, and the change was statistically significant. This study reports that the majority of nurse midwives had low pre-interventional knowledge on home care after CS (52.4%); this decreased to 4.8% after the intervention.

A similar finding was found in the study done by Dhummansur et al., where the protocol regarding prevention of SSI associated with CS was found to be effective in increasing knowledge among nurses; the mean knowledge score of respondents was significantly higher in the post-intervention session as compared to the pre-intervention session (15). Also, the same results were reported in a study done by Elsharkawy et al. on the effect of the Educational Module on Nurses Knowledge and Practices Regarding Prevention of CS-SSI. They found a significant improvement in all participants' mean score knowledge after the educational sessions (16). Further, the study done in Egypt by Ismail et al. in assessing the impact of Enhanced Recovery Pathway application outcomes on nurses and women undergoing CS found an increase in the overall knowledge and practice scores achieved by the maternity nurses (17). Evidence-based and structured health education interventions with demonstrations for health care personnel such as nurse midwives updates their understanding level. In this study, the post-CS home care guide improved nurses' knowledge and may potentially influence their clinical practice'. Evidence from recent literature supports this approach. For instance, a study by Musie et al. (18) underscores that continued professional development among midwives directly contributes to improved maternal care delivery, while Olajubu et al. (19) report similar findings in the context of structured postnatal training interventions. Moreover, in Tanzania, a study done by Faustine et al. showed that health education and simulation intervention increased the knowledge and competence of nurse midwives in the prevention of postpartum hemorrhage (20).

Updated and scientific-based information on post-CS home care after hospital discharge is needed by nurse midwives. This will increase the knowledge of nurse midwives and guide them on the uniform contents of education to provide to post-CS mothers that will assist them in self-care at home.

Remarkably, from this study, the post-CS home care guide was also shown to play an important role in enhancing the knowledge of nurse midwives in delivering home care education to post-CS mothers. This will enhance their confidence in providing the evidence-based and uniform home care education necessary to enhance their safe home care practice that will encourage their recovery. The developed post-CS home care guide has value as a scalable health education tool for home care after CS.

### Strengths and limitations

This study represents an initial and exploratory effort to examine the immediate effect of an educational intervention on improving nurse midwives' knowledge regarding post-CS home care following hospital discharge. A key strength of the study is the use of a pre-tested, structured assessment tool developed directly from a validated and evidence-based home care guide. The tool was reviewed by experts in quantitative research and maternal health, enhancing its content validity and ensuring that it appropriately captured the intended knowledge domains.

However, several limitations should be acknowledged. The study utilized convenience sampling across only two facilities. This non-probability technique may introduce selection bias and limit the representativeness of the sample. As a result, the findings may not be generalizable to all nurse midwives beyond the study setting. Additionally, the study focused on the immediate post-intervention effect and therefore does not provide evidence on knowledge retention or long-term behavioral change in clinical practice. These limitations should be considered when interpreting the results.

## Conclusion

The knowledge level of nurse midwives on home care after CS is generally low. Educating nurse midwives via the developed home care guide and the provision of a leaflet to be used in their discharge counselling will help increase their level of understanding and provide uniform education to post-CS mothers. This will help mothers to have a safe recovery, especially in areas with high CS rates and less in-hospital care.

## Recommendations

This study recommends that nurse midwives be exposed to the newly developed post–caesarean section (post-CS) home care guide. Early exposure is expected to facilitate adoption and integration of the guide into the post-CS care package, particularly during the pre-discharge period, to ensure the provision of standardized and consistent discharge education to post-CS mothers.

Further research is recommended to assess long-term knowledge retention among nurse midwives and to explore their experiences in using the post-CS home care guide in clinical practice.

## Data Availability

The data of this study are under the Muhimbili University of Health and Allied Sciences, they will be available upon requesting to the office of Deputy Vice -Chancellor Academic, research and Consultancy through email; dvcarc@muhas.ac.tz.
